# Computational archaeology of the *Pristionchus pacificus *genome reveals evidence of horizontal gene transfers from insects

**DOI:** 10.1186/1471-2148-11-239

**Published:** 2011-08-15

**Authors:** Christian Rödelsperger, Ralf J Sommer

**Affiliations:** 1 Department for Evolutionary Biology, Max-Planck Institute for Developmental Biology, Spemannstrasse 37, 72076 Tübingen, Germany

## Abstract

**Background:**

The recent sequencing of nematode genomes has laid the basis for comparative genomics approaches to study the impact of horizontal gene transfer (HGT) on the adaptation to new environments and the evolution of parasitism. In the beetle associated nematode *Pristionchus pacificus *HGT events were found to involve cellulase genes of microbial origin and Diapausin genes that are known from beetles, but not from other nematodes. The insect-to-nematode horizontal transfer is of special interest given that *P. pacificus *shows a tight association with insects.

**Results:**

In this study we utilized the observation that horizontally transferred genes often exhibit codon usage patterns more similar to that of the donor than that of the acceptor genome. We introduced GC-normalized relative codon frequencies as a measure to detect characteristic features of *P. pacificus *orphan genes that show no homology to other nematode genes. We found that atypical codon usage is particularly prevalent in *P. pacificus *orphans. By comparing codon usage profiles of 71 species, we detected the most significant enrichment in insect-like codon usage profiles. In cross-species comparisons, we identified 509 HGT candidates that show a significantly higher similarity to insect-like profiles than genes with nematode homologs. The most abundant gene family among these genes are non-LTR retrotransposons. Speculating that retrotransposons might have served as carriers of foreign genetic material, we found a significant local clustering tendency of orphan genes in the vicinity of retrotransposons.

**Conclusions:**

Our study combined codon usage bias, phylogenetic analysis, and genomic colocalization into a general picture of the computational archaeology of the *P. pacificus *genome and suggests that a substantial fraction of the gene repertoire is of insect origin. We propose that the *Pristionchus*-beetle association has facilitated HGT and discuss potential vectors of these events.

## Background

The unique genetic repertoire that allows organisms to adapt to environmental changes and to conquer ecological niches has undergone an enormous evolutionary history. Evolutionary events such as speciation, gene duplication, and loss, accompanied by changes on the single nucleotide level, have generated an overwhelming amount of diversity in all domains of life. In addition to the continuous transfer of genetic material to the subsequent generations by means of inheritance, a number of examples are known where genes are transferred across species borders. Such cases of horizontal gene transfer (HGT) commonly occur in prokaryotes [1,2]. More recently, a number of HGT events have been reported in nematodes [3-6]. The nematode *Pristionchus pacificus *has initially been introduced as a satellite system for comparison to developmental processes of *Caenorhabditis elegans *[7]. *P. pacificus *has a necromenic lifestyle. In the wild, it exists in the form of dauer larvae in association with scarab beetles [8]. Dauer larvae only resume development and become adult worms after the death of the beetle. Initial analysis of the genome sequence identified seven cellulase genes which are of microbial origin [9]. In contrast to cellulases in plant parasitic nematodes, *P. pacificus *cellulases originate from independent HGT events [3,9]. One striking feature of the *P. pacificus *genome is that for more than a third of the 24,231 predicted genes, no homologous sequences exist in any other organism. However, over 50% of these genes show evidence for expression in the form of expressed sequence tag (EST) data [10]. Thus the origin of these so called 'pioneer genes' remains unclear. One open question is whether these genes are *Pristionchus*-specific inventions or whether they have been integrated by means of HGT. The lack of homology to any known protein sequence is an ultimate impediment to any kind of phylogenetic analysis. Therefore, alternative approaches for sequence comparisons are preferable to further characterize these genes.

Early after the initial sequencing of the *Escherichia coli *genome, computational approaches were used to elucidate the molecular archaeology of the *E. coli *genome [1]. This involved the identification of foreign DNA introduced by HGT. Most of the detection methods for so called "alien" DNA search for sequences that differ from the rest of the genome with respect to a certain feature such as dinucleotide distribution [11,12] or codon usage [1,13,14].

In this study we will compare codon usage profiles of *P. pacificus *genes within and across genomes. Focusing on orphan genes that do not show homology to any other nematode sequence, we use dozens of genomewide profiles for nematodes, bacteria, fungi, insects and plants to show that the *P. pacificus *orphan genes are strongly enriched in insect-like codon usage. In cases where homology data is available, genes with predicted insect-like codon usage show a significant association with HGT candidates defined by cross-species homology. In a more detailed analysis of the common HGT candidates, we investigate the role of retrotransposons in the prospective transfer of genetic material from insects to the *P. pacificus *genome.

## Results

### Orphan genes are associated with atypical codon usage

Every genome has a unique pattern of relative synonymous codon usage (RSCU) (see [15] for review). Alternative synonymous codon usage has been associated with tRNA abundances, translational robustness, mRNA secondary structures and genomic GC content. Consistent with this observation, differences across genomes are greater than differences within genomes. Thus recently introduced genes may be identified when their codon usage bears greater resemblance to the donor than the acceptor genome. As a consequence, methods have been developed to detect alien genes with atypical codon usage profiles relative to the genomewide average [1,13,14].

**Figure 1:**
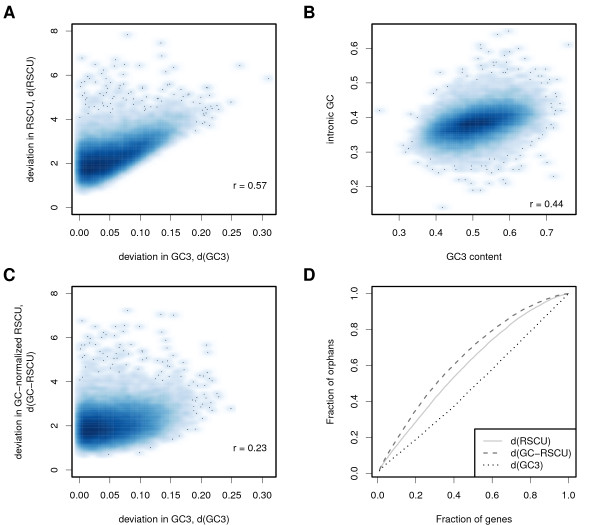
***P. pacificus *orphan genes are enriched in atypical codon usage**. **(A) **For all *P. pacificus *genes with *>*100 codons, RSCU values were computed, and RSCU and GC3 deviation of a single gene from the genomewide average were plotted. Both values show a substantial correlation indicating that RSCU values are strongly biased by GC3 variation. **(B) **Correlation between GC3 and intronic GC content suggests that this bias is due to genomic variation in GC content and not specific for individual transcripts. **(C) **GC-normalization of RSCU values strongly reduces the correlation between deviation in GC3 and codon usage bias. **(D) **Cumulative distribution of *P. pacificus *orphan genes that were ranked by their deviation from the genomewide average of RSCU, GC-normalized RSCU and GC3 content. Genes with most atypical codon usage profiles show an enrichment in orphan genes (*P *= 0.0002), and the strongest enrichment is observed for the GC-normalized RSCU values.

Foremost, we tested if atypical RSCU profiles of *P. pacificus *could be used to detect genes that presumably originate from species outside of the nematode phylum. To do so, we determined whether genes with atypical RSCU profiles are enriched in the set of 9217 *P. pacificus *orphan genes, *i.e*. genes that do not have a homolog in any other nematode species (see *Methods*). One confounding factor in the measurement of atypical codon usage is the genomewide variation of GC content; it has been shown that codon usage in nematodes is largely affected by variations in GC content across different species [16]. To assess the impact of GC content on codon usage we calculated the average RSCU profile for *P. pacificus *and tested whether the Euclidean distance between the genomewide profile and the RSCU profiles of individual genes correlate with differences in GC content of the third codon position (GC3). We indeed found good correlations between deviations in RSCU and GC3 content (*r *= 0.57, Pearson, Figure 1A). In addition, we observed a correlation of *r *= 0.44 between intronic GC content and GC3 content (Figure 1B), suggesting that intraspecies-specific genomic variations in GC content largely affect variations in RSCU profiles for individual genes.

To decrease the impact of GC content on RSCU values, we introduced GC-normalized RSCU values which drop the assumption that all synonymous codons are equally likely. This also takes into account the GC3 content of the whole gene in the calculation of expected codon frequencies (see *Methods*). GC normalization substantially decreases the correlation between RSCU differences and differences in GC3 content to *r *= 0.21 (Figure 1C). Additional file 1 shows the distribution of GC-normalized RSCU deviation from the genomewide profile. We subsequently tested whether the three deviation measures RSCU, GC-normalized RSCU, and GC3 are associated with *P. pacificus *orphan genes. We ranked all genes according to decreasing values of each deviation measure and calculated the fraction of orphans in the set of most atypical genes, as defined by various cutoffs. Figure 1D shows the resulting cumulative distribution of orphan genes with respect to the three measures. In comparison to the deviation in RSCU and GC3, the GC-normalized RSCU measure shows a strong enrichment (up to two-fold for the first 5%) of *P. pacificus *orphan genes (*P *= 0.0002, one-sample KS-test). Differences in non-normalized RSCU yield an enrichment in orphan genes that is two orders of magnitude less significant (*P *= 0.02). In contrast, deviation in GC3 content alone shows no significant enrichment at all (*P *= 1). These results suggest that a large fraction of *P. pacificus *orphan genes show indeed atypical patterns of synonymous codon usage, a trend that cannot be explained by variation in GC3 content.

### Orphan genes show elevated levels of insect-like codon usage

Next we sought to explore if the atypical codon usage could be used to identify the donor through which these genes entered the *P. pacificus *genome. To this end, we compiled a data set of coding sequences totaling 71 bacterial, fungal, amoebozoa, plant, and insect genomes and tested whether orphan genes show a tendency for enrichment in a particular taxonomic group. First, we calculated genomewide GC-normalized RSCU profiles for each species and assigned individual genes to the species with the closest Euclidean distance in RSCU profiles. This "nearest neighbor" approach was able to predict the correct species in 34.4% of the cases and the correct taxonomic group in 71.7% (Table 1). While classification errors exist, they represent a minority of genes and vary across taxonomic groups. For example, it is much more likely that a randomly selected insect gene is mispredicted as a nematode gene (~22% misclassifications, Table 1) than vice versa (~12% misclassifications, Table 1). Given the correct classification of genes in the majority of cases, the nearest neighbor method is well suited to suggest the taxonomic group of a potential donor species for HGT events. In addition to the nearest neighbor approach, we also applied a classification method based on random forests [17] and tested this method on GC-normalized RSCU as well as non-normalized RSCU values and taking into account gene size and GC3 content. However this method did not qualitatively change the results.

**Table 1:** Accuracies of taxonomic group predictions based on GC-normalized codon usage profiles

Taxonomic group	insect*	bacteria*	amoebozoa*	fungi*	nematode*	plant *
Insects	61.1 ± 0.7	6.8 ± 0.5	0.3 ± 0.1	7 ± 0.4	22.0 ± 0.3	2.8 ± 0.2
Bacteria	8.5 ± 0.2	85.5 ± 0.4	0 ± 0	1.9 ± 0.1	3.6 ± 0.2	0.5 ± 0.1
Amoebozoa	2.2 ± 0.6	1.5 ± 0.3	82.9 ± 1.2	1.7 ± 0.7	10.8 ± 1.7	0.9 ± 0.6
Fungi	10.3 ± 0.6	1.3 ± 0.1	0.2 ± 0.1	71.3 ± 0.7	9.1 ± 0.5	7.9 ± 0.3
Nematodes	12.0 ± 0.4	5.5 ± 0.2	0.5 ± 0.1	7.1 ± 0.2	71.9 ± 0.6	3.0 ± 0.2
Plants	6.8 ± 0.8	2.9 ± 0.4	0.3 ± 0.1	18.1 ± 0.6	13.9 ± 0.9	58.0 ± 1.2

Accuracy was measured using ten randomized data sets including equal number of sequences per species (see *Methods*). For each taxonomic group (rows), we calculated the mean percentage of predictions for all possible classifications, the "*" indicates the predicted taxonomic group (columns). For instance, while 61.1% of insect genes are correctly recognized as insect sequences based on their codon usage, 22.0% are mistaken as nematode genes.

**Figure 2:**
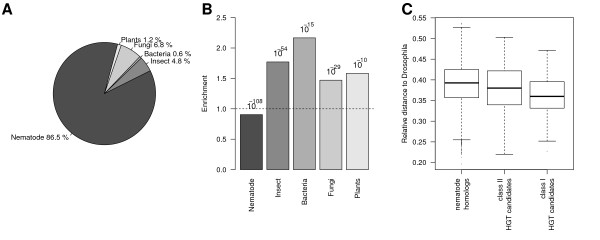
**Prediction of taxonomic groups from codon usage profiles**. **(A) **Distribution of predicted taxonomic groups based on GC-normalized RSCU profiles. Codon usage profiles of *P. pacificus *genes were compared to the average genomewide profiles of 71 species and assigned to the species with the closest Euclidean distance in profiles. **(B) **Enrichment of predicted taxonomic groups for *P. pacificus *orphans with respect to all genes. The strongest enrichment in predicted taxonomic groups is observed for bacteria, however due to the small number of bacteria predictions in general, it is not the most significant enrichment and might be a side effect of the depletion in predicted nematode codon profiles. In contrast, the most significant bias is observed for genes with predicted insect-like codon usage. **(C) **Relative distance to *Drosophila melanogaster *GC-normalized RSCU profile with respect to distances to *P. pacificus *and *Aspergillus nidulans*. In comparison to *P. pacificus *genes with homologs in other nematodes, both classes of HGT candidates show a strongly reduced distance to the *Drosophila melanogaster *profile.

Next, we used the nearest neighbor approach to assign all *P. pacificus *genes to taxonomic groups. Figure 2A shows the distribution of taxonomic group classification results. 86.5% of *P. pacificus *genes are classified as nematode genes. The remaining 13.5% is distributed among fungi (6.8%), insect (4.8%), plants (1.2%), and bacteria (0.6%). This distribution is most likely influenced by the classification error discussed previously; however, the genomewide distribution may serve as a baseline level for comparison with subsets of genes. If we consider only orphan genes, nematode-like codon usage is strongly depleted. Interestingly, the most significant enrichment for orphan genes is observed in insect-like codon usage (*P <*10^-54^, Fisher's exact test). Specifically, 8.5% of the analyzed orphan genes are classified as insect genes. When applied to the whole genome, this value represents 54.7% of all genes that are classified as insect genes and denotes a ~1.8 fold enrichment relative to all genes (Figure 2B).

### HGT candidates defined by cross-species homology are associated with insect-like codon usage

Given the strong enrichment of insect-like codon usage in *P. pacificus *orphan genes, we searched for further evidence of HGT from insects by comparing the results obtained from our codon usage analysis to HGT candidates defined by cross-species homology. We compiled a database including nematode as well as insect protein sequences to test whether HGT candidates defined by cross-species homology show a significant association with insect-like codon usage. We searched the blastp results for two classes of genes that would give rise to HGT candidates. First, we looked for *P. pacificus *orphan genes that had no homologous sequence in other nematodes but had a blastp hit (*e*-value *<*0.001) in insects (class I). Second, we scanned for genes with homologs in other nematodes but with a minimum thousand fold smaller blastp *e*-value for a hit against any insect protein (class II). We identified 205 class I and 304 class II HGT candidates. The 509 HGT candidates with best blastp hits, taxonomic group predictions, and PFAM domains are presented in additional file 2.

Most of the HGT candidates were predicted to be nematode genes based on their codon usage, *i.e*. 76.5% of class I and 84.7% of class II HGT candidates. This result indicates that the majority of genes acquired by HGT have been adapted to the codon usage of the host genome, an observation that has also been suggested by Karlin et al. [13]. Alternatively, this finding might be due to the similarity between the codon usage profiles of the donor and host genomes.

However, for the remaining HGT candidates, evidence for the taxonomic group of donors can be obtained. 9% of class II HGT candidates exhibit insect-like codon usage, which represents a significant 1.9 fold enrichment relative to the fraction of predicted insect-like codon usage among all genes (*P <*10^-5^, Fisher's exact test). For the 205 class I candidates there was no enrichment in insect-like codon usage, but a 1.3 fold enrichment in predicted fungal profiles (see *Discussion*) which is in contrast to the 9217 orphan genes (see above). To test whether both classes of HGT candidates show a higher similarity to insect-like codon usages than genes with nematode homologs, we considered relative rather than absolute distances of single genes to genomewide profiles of nematodes, fungi, and insects. In particular, we used the distance relative to the sum of nematode, fungal and insect distances, where *Drosophila *and *Aspergillus *represent insects and fungi, respectively. We found that both HGT candidate classes show a significant reduction in distance to the *Drosophila *profile (*P <*10^-13 ^for class I candidates and *P <*0.01 for class II candidates, Wilcoxon test, Figure 2C).

These results suggest, that despite the close similarity between insect, fungal, and nematode codon usages, HGT candidates defined by cross-species homology show a significantly increased similarity to insect-like codon usages. Taken together, the genomewide computational analysis of HGT candidates defined by cross-species homology supports an association with insect-like codon usage.

### Phylogenetic analysis reveals integration of non-LTR retrotransposons

**Figure 3:**
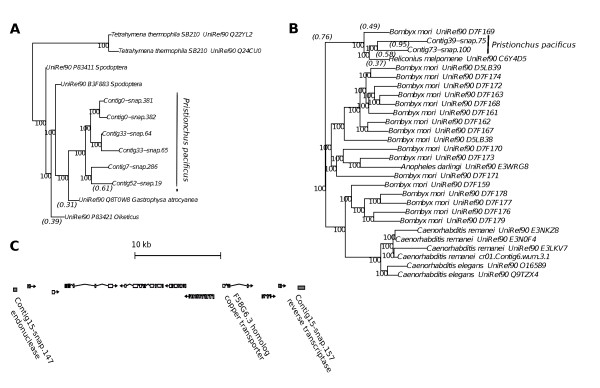
**Phylogenetic relationships for P. pacificus Diapausins and reverse transcriptases**. **(A) **Maximum likelihood tree of Diapausin genes including six *P. pacificus *HGT candidates. Node labels indicate bootstrap support values (100 replications). All *P. pacificus *Diapausins show the closest similarity to a sequence from leaf beetle (*Gastrophysa atrocyanea*). **(B) **Phylogenetic tree of reverse transcriptase proteins from *P. pacificus *and other nematode and insect sequences. The two *P. pacificus *reverse transcriptases show highest similarity to a sequence from the postman butterfly (*Heliconius melpomene*). **(C) **Genes in a 40 kb locus on Contig15 of the *P. pacificus *Hybrid 1 assembly. The locus is bounded by an endonuclease and a reverse transcriptase and contains eight embedded genes. Seven of these genes do not show homology to any other known sequences. The only non-orphan gene is a copper transporter with homology to *C. elegans *F58G6.3.

To elucidate what genes could have been acquired by *P. pacificus*, we looked more closely into the gene sets identified by cross-species homologies. First, we used the previously identified Diapausin gene as a control [9]. Diapausins encode diapause-specific peptides that provide antifungal activity by acting as Ca2+ channel blockers [18]. Among the HGT candidates supported by cross-species homologies and predicted insect-like codon usage, we indeed identified two genes that showed closest similarity to Diapausins from *Spodoptera *and *Gastrophysa*. The *P. pacificus *genome contains four other members of this gene family (PF08036.4) which are all among the HGT candidates defined by cross-species homology; Figure 3A shows a phylogenetic tree of this gene family. All *P. pacificus *Diapausin genes originate from a common sequence and show closest similarity to the leaf beetle Diapausin. This positive control confirms the effectiveness of our approach towards the identification of HGT candidates.

Table 2 shows the 20 most abundant PFAM domains that were found in the set of 509 HGT candidates defined by cross-species homology. The two most abundant domains correspond to a reverse transcriptase (PF00078.20) and endonuclease domain (PF03372.16). Both domains also occur in HGT candidates that are supported by cross-species homologies and predicted insect-like codon usage. These genes are remote homologs of the *C. elegans *retrotransposable element Rte-1 [19], a member of the non-LTR retrotransposable elements. Rte-1 encodes an endonuclease and a reverse transcriptase enclosed by target site duplication sequences at the 5' and 3' end. Similar examples of a Rte-1-like HGT events have been reported from plants to fish and from arthropods to reptiles [20]. A more recent phylogenetic analysis observed a greater similarity of a *P. pacificus *reverse transcriptase to that of *Bombyx mori *sequences without any further discussion [21].

**Table 2:** Pfam domains in HGT candidates

Accession ID	Description	*N*	Enrichment	*p*-value
PF00078.20	Reverse transcriptase (RNA-dependent DNA polymerase)	58	14.0×	*<*10^-49^
PF03372.16	Endonuclease/Exonuclease/phosphatase family	27	18.8×	*<*10^-26^
PF00096.19	Zinc finger, C2H2 type	14	5.1×	*<*10^-5^
PF00067.15	Cytochrome P450	11	2.5×	*<*0.01
PF00069.18	Protein kinase domain	9	0.9×	1
PF07714.10	Protein tyrosine kinase	9	1.0×	0.9
PF00097.18	Zinc finger, C3HC4 type (RING finger)	8	3.1×	*<*0.01
PF00089.19	Trypsin	6	6.4×	*<*0.01
PF08036.4	Diapausin family of antimicrobial peptide	6	38.3×	*<*10^-8^
PF08953.4	Domain of unknown function (DUF1899)	6	25.6×	*<*10^-6^
PF00076.15	RNA recognition motif (RRM)	5	1.6×	0.3
PF00644.13	Poly(ADP-ribose) polymerase catalytic domain	5	24.0×	*<*10^-5^
PF00856.21	SET domain	5	5.8×	*<*0.01
PF03732.10	Retrotransposon gag protein	5	24.0×	*<*10^-5^
PF12171.1	Zinc-finger double-stranded RNA-binding	5	3.8×	*<*0.05
PF00092.21	von Willebrand factor type A domain	4	3.2×	0.06
PF00106.18	short chain dehydrogenase	4	1.4×	0.4
PF00178.15	Ets-domain	4	8.5×	*<*0.01
PF00005.20	ABC transporter	3	1.4×	0.6
PF00098.16	Zinc knuckle	3	3.7×	*<*0.05

The 20 most abundant Pfam domains (*e*-value *<*0.001) in 509 HGT candidates defined by cross-species homology are shown. Enrichments factors and FDR corrected *p*-values were calculated relative to all *P. pacificus *genes with annotated PFAM domains.

In total, the *P. pacificus *genome encodes 159 genes with a strong similarity to reverse transcriptase domains (*e*-value *<*0.001). 70 of these domains exhibit a higher similarity to a reverse transcriptase from insects than to the closest homolog within the nematode phylum. The remaining reverse transcriptase domains have closest homologs in other nematodes. Together, these findings indicate that the *P. pacificus *genome contains ancient homologs of the *C. elegans *Rte-1 elements as well as copies that might have originated from insects. Figure 3B shows a phylogenetic tree of reverse transcriptase proteins including two *P. pacificus *genes with closest homologs in insects.

### Orphan genes and HGT candidates preferentially colocalize near retrotransposons

The HGT candidates supported by cross-species homologies and predicted insect-like codon usage contain, in addition to the non-LTR retrotransposons, a second retrotransposon associated gene. This gene shows highest similarity to a gene prediction encoding a gag protein of a retrotransposon (PF03732.10, *e*-value *<*10^-16^) from the red flower beetle *Tribolium castaneum *(see additional file 2). Gag proteins are known to mediate the telomer-specific transposition of retrotransposons for telomer maintenance in *Drosophila *[22].

The discovery of retrotransposons in HGT candidates led us to wonder whether retrotransposons are not only detectable outcomes of HGT events, but are possible mediators of HGT by cotransposition of intervening genes. A number of studies reported transfers of genetic material across species borders by means of molecular parasites. These reports include the transfer of *P *elements between *Drosophila *species [23] and a transfer of a fungal endonuclease gene into an intron of the coxl gene in the plant *Peperomia polybotrya *[24] (see [25] for review).

Therefore, we searched for endonuclease and reverse transcriptase bounded intervals of up to 40 kb in the *P. pacificus *genome. We identified one prominent example that encompasses 10 genes, which are bounded by an endonuclease and a reverse transcriptase in the same orientation (Figure 3C). Out of the eight intervening genes, only one is found in other nematodes, whereas no homologous sequence could be detected for the remaining ones. This suggests that retrotransposons are able to carry more than just the genes required for transposon activity.

Next, we tested for a genomewide tendency of orphan genes to cluster in the vicinity of retrotransposons and searched for significant enrichment of orphan genes and class II HGT candidates in their vicinity. We first defined a reference set of all retrotransposon genes (reverse transcriptases (PF00078.20), endonucleases (PF03372.16), and gag proteins (PF03732.10)) with higher similarity to insects than to nematodes. We then repeatedly sampled non-overlapping genomic locations of equal number and size distribution as that of the merged 30 kb upstream and downstream regions of the retrotransposon genes. In total, these regions span 5.2 Mb and encompass 352 orphan genes and class II HGT candidates, representing a 1.15 ± 0.08 fold enrichment relative to randomly sampled genomic locations (*P *= 0.034, 1000 iterations). Thus, HGT candidates nonetheless show a tendency to cluster around retrotransposon genes. However, we could not detect any significant trend towards either upstream or downstream regions of reverse transcriptases and endonucleases. These findings are in agreement with the assumption that insect retrotransposons might have been used as vectors for integration of foreign DNA into the *P. pacificus *genome.

### HGT candidates defined by cross-species homology are conserved in the genus Pristionchus

An association with retrotransposons suggests that more or less random genetic material has been integrated into the *P. pacificus *genome. For evolutionary significance of HGT events, integration into the host biology and permanence are required [26]. Even if foreign DNA can be integrated into a host genome, it will be rapidly degraded by neutral evolution, unless it will prove beneficial for the host. To test, whether the HGT candidates have been introduced into the *P. pacificus *genome only recently, we searched for homologous sequences in the low coverage genomes of *Pristionchus entomophagus *and *Pristionchus maupasi *(blastn *e*-value *<*0.001) [9]. We found homologous sequences for 218 (41.6%) of HGT candidates in at least one other *Pristionchus *species, 105 (20.6%) of HGT candidates showed evidence for conservation in both species, indicating that a substantial fraction of HGT candidates date back to ancestral sequences within the genus *Pristionchus*. However, more data will be needed to evaluate whether these genes evolve neutrally or are under any kind of evolutionary constraint.

## Discussion

Novel sequencing technologies have dramatically increased the number of available genome sequences over the last few years. However, the biological value of a new genome sequence is limited due to the lack of knowledge about homologous sequences in other organisms. The absence of homology to any known sequence, as in the case for a large fraction of *P. pacificus *genes [9,10], exemplifies our lack of knowledge about the genome content of this model organism. Therefore, we have started to complement the available *P. pacificus *genome, transcriptome and proteome data with next generation sequencing approaches of related species [3,9]. This approach has facilitated a mechanistic understanding of some of the HGT events that occurred in the evolutionary lineage giving rise to *P. pacificus*. For cellulase genes acquired from microbes, *P. pacificus *and related *Pristionchus *nematodes show functional assimilation, high gene turnover and rapid sequence diversification associated with positive selection [3].

In *P. pacificus*, the scarab beetle-associated ecology might result in a number of potential donors for HGT. The decaying beetle is an ecosystem consisting of bacteria, fungi, nematodes and presumably, a large number of unicellular eukaryotes. The previously described examples of cellulase and Diapausin genes clearly indicate that microbes and insects, at least, must be considered as potential HGT donors into the *P. pacificus *genome. One inroad into the identification of HGT events is a computational archaeology approach as originally described for *E. coli *[1].

In this study we have hypothesized that a substantial fraction of the *P. pacificus *orphans might be introduced into the genome by means of HGT. Hereby we refer to an orphan gene as a gene with no similarity to any other nematode sequence. Under the assumption that some horizontally transferred genes may exhibit a codon usage bias that is more similar to the donor genome than to the acceptor genome [1,13], we could show that a fraction of *P. pacificus *orphans exhibits an atypical codon usage relative to the rest of the genome. The fact that the majority of orphan genes show a codon usage typical for nematodes might be due to two circumstances. First, HGT events most likely occurred repeatedly with more recent HGT events preferentially showing a codon usage bias. Second, with multiple potential donors, no common patterns of atypical codon usage are expected. For example, nematodes, insects and fungi show closely related codon usage patterns, whereas protozoans and other microbes, all of which are potential donors for HGT, exhibit very different codon usages. In our analysis, we found a similarity in codon usages for insects, nematodes and fungi. GC-normalized RSCU distances of *P. pacificus *genes to the genomewide profiles of *P. pacificus*, *Drosophila melanogaster*, and *Aspergillus nidulans *showed strong correlations (*r >*0.87, Pearson). This circumstance highlights the need for a careful investigation of potential HGT events. We consider the work presented in this study as a novel computational entry road towards the identification of HGT patterns in *P. pacificus*.

In addition to the strong association of orphan genes with atypical codon usage, we could characterize this codon usage pattern by comparison to genomewide profiles for 71 species corresponding to six taxonomic groups. The extent to which codon usage profiles can predict species and taxonomic groups is still limited. However, comparisons of subsets of genes against all genes may help uncover the domain or phylum, from which these genes entered the *P. pacificus *genome. The most significant enrichment was detected for insect-like codon usage (*P <*10^-54^).

It is important to note that atypical patterns of codon usage may also arise from other sources such as translational efficiency or secondary structures (see [15] for review). Thus analysis of codon usage alone may not be sufficient to support the proposed HGT events. We therefore complemented this analysis by cross-species comparisons to identify genes that show greatest similarity to homologs in insects.

We identified 509 HGT candidates using homology searches against a combined nematode and insect protein database and scanning for genes bearing greater resemblance to insect genes than to the closest homologs within the nematode phylum. These HGT candidates showed a significantly higher similarity to insect-like codon usage profiles. Further investigations revealed that in addition to the previously identified Diapausins (Table 2) [9], many of these genes encode endonuclease and reverse transcriptase proteins. Since 70 of the 159 *P. pacificus *reverse transcriptase sequences show a higher degree of similarity to those of insects, we speculate that reintroduction of these elements from insects represents one mechanism by which *P. pacificus *has acquired genes. Phylogenetic analysis of all HGT candidates identified by cross-species homology could provide more detailed information and further support for the proposed HGT events. Although *P. pacificus *is not an insect parasitic nematode, dauer larvae of *P. pacificus *are in constant physical contact with beetles [27]. After the death of the beetle, nematodes resume development and feed on microorganisms growing on the carcass, presumably for several generations [28]. Close physical contact between donor and recipient has been proposed as one criteria for HGT [5], making beetles a plausible candidate for HGT donors. While our data suggests that a substantial fraction of *P. pacificus *orphans originates from insect genomes, it is possible that HGT involves vectors as intermediate carriers. It is known that many viruses coexist with insects often in a species-specific interaction, so viruses are obvious candidates for HGT into *P. pacificus*. This hypothesis is supported by the finding that parts of the Diapausin genes found in leaf beetles and *P. pacificus *have also been observed in iridoviruses [18]. We therefore hypothesize that viruses are potential intermediate carriers that promote HGT events from insects into *P. pacificus*.

Our data however, strongly support a second scenario. We identified a large number of non-LTR retrotransposon sequences in the *P. pacificus *genome that have highest sequence similarity to insects. In addition to permanence, integration into the host's biology is one necessary features of HGT [26]. The non-LTR retrotransposons are unlikely to have a beneficial effect on the biology of *P. pacificus*. Thus the strong enrichment of retrotransposon associated genes among HGT candidates defined by cross-species homology seems counterintuitive. One explanation for this observation is that retrotransposons might have served as carriers of foreign genetic material into the *P. pacificus *genome. This hypothesis is supported by the fact that we detected a tendency for orphan genes and other HGT candidates to be colocalized near retrotransposon genes. It could provide one possible explanation for the presence of foreign retrotransposons and could serve as a model for how other genes might have integrated into the *P. pacificus *genome. An open question is the permanence of the transferred genetic material. Comparison with other *Pristionchus *species indicates, that a substantial fraction of HGT candidate dates back to ancestral *Pristionchus *sequences. However, more data from wild isolates will be needed to robustly measure the amount of selection acting on these genes.

## Conclusions

The computational archaeology of the *P. pacificus *genome combines analysis of codon usage bias with phylogenetic analysis, both of which reveal evidence of HGT events from insects. We identified colocalized gene clusters surrounded by non-LTR retrotransposons, suggesting a mechanism of HGT that involves transposable elements [23,24]. This study highlights that multiple computational approaches are necessary to obtain an overview of HGT and other potential genomewide mechanisms contributing to the evolution of eukaryotic genomes.

## Methods

### Identification of orphan genes

We used the gene predictions for 24,231 *Pristionchus pacificus *genes [10] and downloaded gene annotations for 30,163 *Caenorhabditis elegans *transcripts (WS220), 21,991 *Caenorhabditis briggsae *transcripts (WS223), and 21,332 from *Brugia malayi *transcripts (WS222).

We compiled a protein sequence database for all non-*Pristionchus *nematode protein sequences which included 20,359 *Meloidogyne incognita *proteins obtained from the *M. incognita *resources website [29], 157,761 protein translations of non-*P. pacificus *nematode ESTs from nematode.net [30] and non-*Pristionchus *nematode sequences from the UniRef90 database. We then searched this database for *P. pacificus *homologs using NCBI blastp (version 2.2.22) [31].

For 15,014 (62.0%) of *P. pacificus *genes, at least one nematode homolog with *e*-value *<*0.001 could be identified. This translates to 9217 (38.0%) orphan genes.

### GC content and codon usage profiles

To eliminate biases due to recent gene expansions or alternatively spliced transcripts in the genomewide calculation of RSCU profiles, we clustered all transcripts with ≥ 80% sequence identity using cd-hit-est [32] (version 4.3 with -c 0.8 options).

We further restricted the analysis of codon usage to only those transcripts with at least 100 codons. This reduced the number of *P. pacificus *transcripts to 19,515, 6370 of which are in the set of orphan genes. For each transcript with at least 100 codons, we determined the GC3 content for the whole gene and then calculated the GC-normalized RSCU values for a codon *x *as

(1) $$RSCU_{GC}\left(x\right)=\frac{f_{ob}\left(x\right)}{f_{exp}\left(x\right)},$$

where *f_ob_*(*x*) denotes the observed frequency of codon *x *among all synonymous codons and *f_exp_*(*x*) is calculated as

(2) $$f_{exp}\left(x\right)=\frac{p\left(x|GC3\right)}{\sum_{y\in syn\left(x\right)}p\left(y|GC3\right)},$$

where *syn*(*x*) denotes the set of all synonymous codons for *x *and *p*(*x*|*GC*3) indicates the probability of codon *x *for a given GC3 content. *p*(*x*|*GC*3) is calculated as the product of the three individual nucleotide probabilities given the GC3 content

(3) $$p\left(x|GC3\right)=GC3^{k}\times {\left(1-GC3\right)}^{3-k},$$

whereby *k *denotes the number of nucleotides that are either G or C. Deviation in RSCU values for single genes was computed as the Euclidean distance to the average genomewide RSCU profile. Analogously deviation in GC3 for a single gene was calculated as the absolute difference from the genomewide average GC3 content.

### Transcriptome and EST data

We downloaded protein coding transcript annotations for 15 bacterial and 12 fungal genomes from Ensembl Bacterial and Fungal Mart (Release 8), and unique representative EST sequences from UniGene for 5 plant, 8 insect, and 1 amoebozoa species. These representative sequences were defined as the sequence with the longest region of high-quality sequence data for each UniGene EST cluster. In addition we downloaded nematode consensus EST sequences from nematode.net [30,33].

For each EST sequence we identified the coding sequence (CDS) as the longest sequence with no stop codons among all 6 potential reading frames; for that sequence, the transcript could start either at the first codon or at the last ATG. If the longest sequence was at least 300 bp long and the second longest was at most 150 bp long, we used this CDS for further analysis.

We obtained 17,189 transcripts for red harvester ant *Pogonomyrmex barbatus *(Gene Set v1.2) from the Hymenoptera Genome Database [34], 21,899 *Drosophila melanogaster *transcripts from the Ensembl 61 database, 14,623 transcripts for silkworm *Bombyx mori *from SilkDB [35], 11,062 transcripts for honey bee *Apis mellifera *(PreRelease2 OGS) from BeeBase [34], and 18,249 sequences for red flour beetle *Tribolium castaneum *from the Tribolium castaneum Genome Project website (Tcas2.0) [36].

### Prediction of species and taxonomic groups based on RSCU profiles

To predict species given the GC-normalized RSCU values of a single gene, we compared the RSCU profile of the gene with genomewide profiles for all species and assigned it to the nearest neighbor, which we defined as the species with the closest Euclidean distance between RSCU profiles.

The accuracy of the nearest neighbor method for species and taxonomic group predictions was calculated as the fraction of correct predictions among all predictions. The reported accuracies are based on ten evaluations of data sets for 71 species (13 insects, 15 bacteria, 25 nematodes, 12, fungi, 5 plants and 1 amoebozoa) with more than 1000 sequences containing greater than 100 codons. From each species 333 sequences were randomly drawn and put into the validation set.

### Identification of HGT candidates by cross-species homology and phylogenetic analysis

To define HGT candidates we combined the nematode proteins with protein translations for the five insect genomes and insect and arthropod sequences from the UniRef90 data base. We searched for class I and II HGT candidates in the resulting set of 721,041 protein sequences using blastp.

For the non-LTR retrotransposon reverse transcriptase we used the program hmmsearch from the HMMer package (version 3.0) to scan the nematode and insect protein sequences for presence of a reverse transcriptase (1239 PF00078.20 hits with *e*-value *<*10^-10^) domain. This set was extended by eight *P. pacificus *reverse transcriptases supported by cross-species homologies and predicted insect-like codon usage. To reduce the number of reverse transcriptase protein sequences that were used for the multiple alignment, we clustered the sequences with cdhit (60% identity cutoff) and Transclust [37]. Only one cluster contained *P. pacificus *HGT candidates and other reverse transcriptases. We then used muscle (version 3.8.31, [38]) to align sequences and the phangorn R package [39] to estimate a maximum-likelihood tree under the LG amino acid substitution model [40] with the Gamma + I model to account for rate variation across sites [41]. For Diapausins we scanned the whole UniRef90 database for Diapausin domains (six PF08036.4 hits with *e*-value *<*10^-3^). This set was extended by six *P. pacificus *Diapausins and subsequently used for alignment and maximum-likelihood tree estimation.

## Authors' contributions

CR and RJS conceived the work. CR analyzed the data. CR and RJS wrote the paper. Both authors read and approved the final manuscript.

## Supplementary Material

Additional file 1 **Distribution of GC-normalized RSCU deviation from the genomewide profile**. Histogram of Euclidean distances between GC-normalized RSCU values of single genes and the genomewide profile of *P. pacificus*. For all orphans and non orphans, the frequency of genes in each Euclidean distance bin is shown. Click here for file

Additional file 2 **List of HGT candidates defined by cross-species homology**. List of HGT candidates with best blastp hit, predicted taxonomic group based on codon usage, and identified PFAM domains. Click here for file

## References

[B1] Lawrence JG Ochman H Molecular archaeology of the Escherichia coli genome Proc Natl Acad Sci USA 1998 95 16 9413 9417 10.1073/pnas.95.16.9413 9689094 PMC21352

[B2] Boucher Y Douady CJ Papke RT Walsh DA Boudreau MER Nesbø CL Case RJ Doolittle WF Lateral gene transfer and the origins of prokaryotic groups Annu Rev Genet 2003 37 283 328 10.1146/annurev.genet.37.050503.084247 14616063

[B3] Mayer WE Schuster LN Bartelmes G Dieterich C Sommer RJ Horizontal gene transfer of microbial cellulases into nematode genomes is associated with functional assimilation and gene turnover BMC Evol Biol 2011 11 13 10.1186/1471-2148-11-13 21232122 PMC3032686

[B4] Danchin EGJ Rosso MN Vieira P de Almeida-Engler J Coutinho PM Henrissat B Abad P Multiple lateral gene transfers and duplications have promoted plant parasitism ability in nematodes Proc Natl Acad Sci USA 2010 107 41 17651 17656 10.1073/pnas.1008486107 20876108 PMC2955110

[B5] Mitreva M Smant G Helder J Role of horizontal gene transfer in the evolution of plant parasitism among nematodes Methods Mol Biol 2009 532 517 535 10.1007/978-1-60327-853-9_30 19271205

[B6] Hotopp JCD Clark ME Oliveira DCSG Foster JM Fischer P Torres MCM Giebel JD Kumar N Ishmael N Wang S Ingram J Nene RV Shepard J Tomkins J Richards S Spiro DJ Ghedin E Slatko BE Tettelin H Werren JH Widespread lateral gene transfer from intracellular bacteria to multicellular eukaryotes Science 2007 317 5845 1753 1756 10.1126/science.1142490 17761848

[B7] Sommer RJ Evolution of development in nematodes related to C. elegans WormBook 2005 1 17 10.1895/wormbook.1.46.1 PMC4781599 18050392

[B8] Herrmann M Mayer WE Sommer RJ Nematodes of the genus Pristionchus are closely associated with scarab beetles and the Colorado potato beetle in Western Europe Zoology (Jena) 2006 109 2 96 108 10.1016/j.zool.2006.03.001 16616467

[B9] Dieterich C Clifton SW Schuster LN Chinwalla A Delehaunty K Dinkelacker I Fulton L Fulton R Godfrey J Minx P Mitreva M Roeseler W Tian H Witte H Yang SP Wilson RK Sommer RJ The Pristionchus pacificus genome provides a unique perspective on nematode lifestyle and parasitism Nat Genet 2008 40 10 1193 1198 10.1038/ng.227 18806794 PMC3816844

[B10] Borchert N Dieterich C Krug K Schütz W Jung S Nordheim A Sommer RJ Macek B Proteogenomics of Pristionchus pacificus reveals distinct proteome structure of nematode models Genome Res 2010 20 6 837 846 10.1101/gr.103119.109 20237107 PMC2877580

[B11] Karlin S Global dinucleotide signatures and analysis of genomic heterogeneity Curr Opin Microbiol 1998 1 5 598 610 10.1016/S1369-5274(98)80095-7 10066522

[B12] Arvey AJ Azad RK Raval A Lawrence JG Detection of genomic islands via segmental genome heterogeneity Nucleic Acids Res 2009 37 16 5255 5266 10.1093/nar/gkp576 19589805 PMC2760805

[B13] Karlin S Mrázek J Campbell AM Codon usages in different gene classes of the Escherichia coli genome Mol Microbiol 1998 29 6 1341 1355 10.1046/j.1365-2958.1998.01008.x 9781873

[B14] Merkl R SIGI: score-based identification of genomic islands BMC Bioinformatics 2004 5 22 10.1186/1471-2105-5-22 15113412 PMC394314

[B15] Plotkin JB Kudla G Synonymous but not the same: the causes and consequences of codon bias Nat Rev Genet 2011 12 32 42 10.1038/nrg2899 21102527 PMC3074964

[B16] Cutter AD Wasmuth JD Blaxter ML The evolution of biased codon and amino acid usage in nematode genomes Mol Biol Evol 2006 23 12 2303 2315 10.1093/molbev/msl097 16936139

[B17] Breiman L Random Forests Mach Learn 2001 45 5 32 10.1023/A:1010933404324

[B18] Tanaka H Sato K Saito Y Yamashita T Agoh M Okunishi J Tachikawa E Suzuki K Insect diapause-specific peptide from the leaf beetle has consensus with a putative iridovirus peptide Peptides 2003 24 9 1327 1333 10.1016/j.peptides.2003.07.021 14706547

[B19] Youngman S van Luenen HG Plasterk RH Rte-1, a retrotransposon-like element in Caenorhabditis elegans FEBS Lett 1996 380 12 1 7 8603714 10.1016/0014-5793(95)01525-6

[B20] Zupunski V Gubensek F Kordis D Evolutionary dynamics and evolutionary history in the RTE clade of non-LTR retrotransposons Mol Biol Evol 2001 18 10 1849 1863 11557792 10.1093/oxfordjournals.molbev.a003727

[B21] Tay WT Behere GT Batterham P Heckel DG Generation of microsatellite repeat families by RTE retrotransposons in lepidopteran genomes BMC Evol Biol 2010 10 144 10.1186/1471-2148-10-144 20470440 PMC2887409

[B22] Rashkova S Karam SE Kellum R Pardue ML Gag proteins of the two Drosophila telomeric retrotransposons are targeted to chromosome ends J Cell Biol 2002 159 3 397 402 10.1083/jcb.200205039 12417578 PMC2173066

[B23] Daniels SB Peterson KR Strausbaugh LD Kidwell MG Chovnick A Evidence for horizontal transmission of the P transposable element between Drosophila species Genetics 1990 124 2 339 355 2155157 10.1093/genetics/124.2.339 PMC1203926

[B24] Vaughn JC Mason MT Sper-Whitis GL Kuhlman P Palmer JD Fungal origin by horizontal transfer of a plant mitochondrial group I intron in the chimeric CoxI gene of Peperomia J Mol Evol 1995 41 5 563 572 7490770 10.1007/BF00175814

[B25] Schaack S Gilbert C Feschotte C Promiscuous DNA: horizontal transfer of transposable elements and why it matters for eukaryotic evolution Trends Ecol Evol 2010 25 9 537 546 10.1016/j.tree.2010.06.001 20591532 PMC2940939

[B26] Blaxter M Symbiont genes in host genomes: fragments with a future? Cell Host Microbe 2007 2 4 211 213 10.1016/j.chom.2007.09.008 18005738

[B27] Weller AM Mayer WE Rae R Sommer RJ Quantitative assessment of the nematode fauna present on Geotrupes dung beetles reveals species-rich communities with a heterogeneous distribution J Parasitol 2010 96 3 525 531 10.1645/GE-2319.1 20557197

[B28] Mayer MG Sommer RJ Natural variation in Pristionchus pacificus dauer formation reveals cross-preference rather than self-preference of nematode dauer pheromones Proc Biol Sci 2011 10.1098/rspb.2010.2760 PMC3145190 21307052

[B29] Abad P Gouzy J Aury JM Castagnone-Sereno P Deleury E Perfus-Barbeoch L Anthouard V Artiguenave F Blok VC Caillaud MC Genome sequence of the metazoan plant-parasitic nematode Meloidogyne incognita Nat Biotechnol 2008 26 8 909 915 10.1038/nbt.1482 18660804

[B30] Martin J Abubucker S Wylie T Yin Y Wang Z Mitreva M Nematode.net update 2008: improvements enabling more efficient data mining and comparative nematode genomics Nucleic Acids Res 2009 37 Database D571 D578 10.1093/nar/gkn744 PMC2686480 18940860

[B31] Altschul SF Gish W Miller W Myers EW Lipman DJ Basic local alignment search tool J Mol Biol 1990 215 3 403 410 2231712 10.1016/S0022-2836(05)80360-2

[B32] Li W Godzik A Cd-hit: a fast program for clustering and comparing large sets of protein or nucleotide sequences Bioinformatics 2006 22 13 1658 1659 10.1093/bioinformatics/btl158 16731699

[B33] Parkinson J Mitreva M Whitton C Thomson M Daub J Martin J Schmid R Hall N Barrell B Waterston RH McCarter JP Blaxter ML A transcriptomic analysis of the phylum Nematoda Nat Genet 2004 36 12 1259 1267 10.1038/ng1472 15543149

[B34] Munoz-Torres MC Reese JT Childers CP Bennett AK Sundaram JP Childs KL Anzola JM Milshina N Elsik CG Hymenoptera Genome Database: integrated community resources for insect species of the order Hymenoptera Nucleic Acids Res 2011 39 Database D658 D662 10.1093/nar/gkq1145 PMC3013718 21071397

[B35] Duan J Li R Cheng D Fan W Zha X Cheng T Wu Y Wang J Mita K Xiang Z Xia Q SilkDB v2.0: a platform for silkworm (Bombyx mori) genome biology Nucleic Acids Res 2010 38 Database D453 D456 10.1093/nar/gkp801 PMC2808975 19793867

[B36] Consortium TGS The genome of the model beetle and pest Tribolium castaneum Nature 2008 452 7190 949 955 10.1038/nature06784 18362917

[B37] Wittkop T Emig D Truss A Albrecht M Böcker S Baumbach J Comprehensive cluster analysis with Transitivity Clustering Nat Protoc 2011 6 3 285 295 10.1038/nprot.2010.197 21372810

[B38] Edgar RC MUSCLE: multiple sequence alignment with high accuracy and high throughput Nucleic Acids Res 2004 32 5 1792 1797 10.1093/nar/gkh340 15034147 PMC390337

[B39] Schliep KP phangorn: phylogenetic analysis in R Bioinformatics 2011 27 4 592 593 10.1093/bioinformatics/btq706 21169378 PMC3035803

[B40] Le SQ Gascuel O An improved general amino acid replacement matrix Mol Biol Evol 2008 25 7 1307 1320 10.1093/molbev/msn067 18367465

[B41] Yang Z Maximum likelihood phylogenetic estimation from DNA sequences with variable rates over sites: approximate methods J Mol Evol 1994 39 3 306 314 10.1007/BF00160154 7932792

