# Supplementary figures and images for: Computational archaeology of the Pristionchus pacificus genome reveals evidence of horizontal gene transfers from insects
Source: BMC Evol Biol. 2011 Aug 15;11:239. doi: 10.1186/1471-2148-11-239 (PMC3175473; doi:10.1186/1471-2148-11-239)

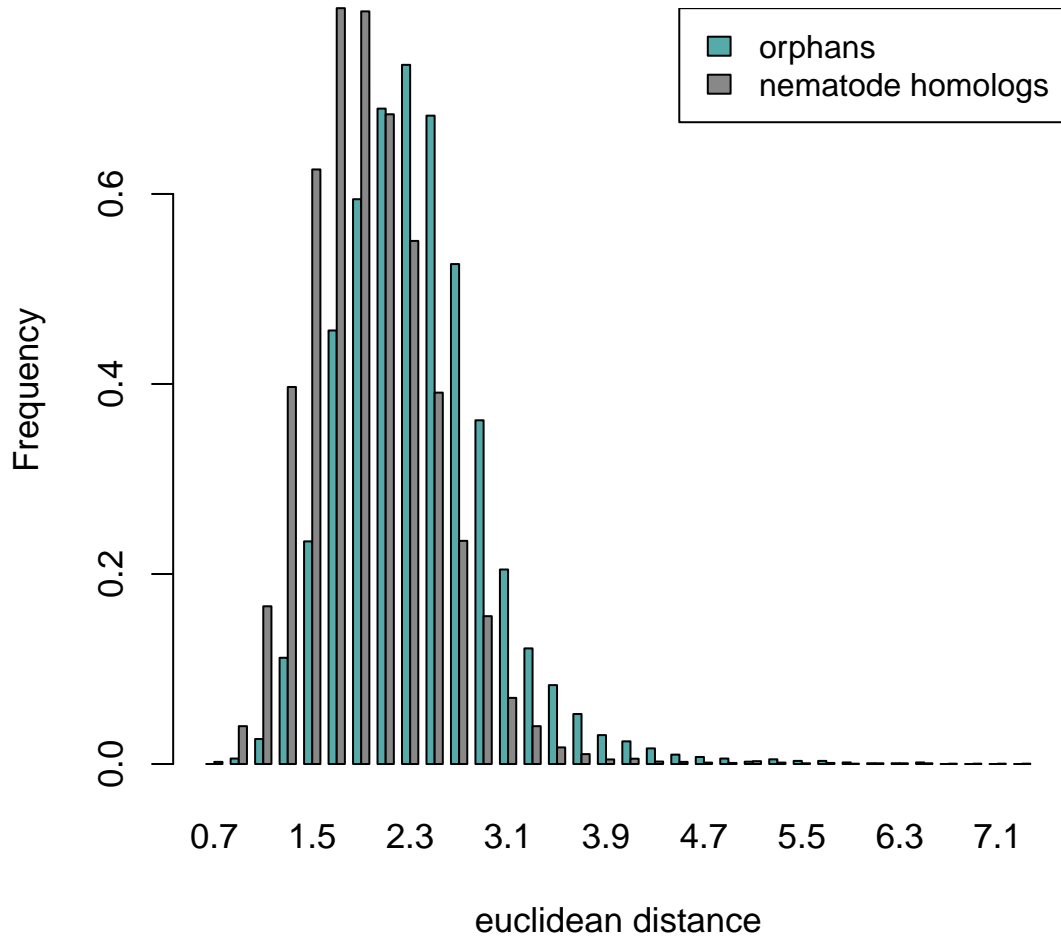

Supplement: Additional file 1 — Distribution of GC-normalized RSCU deviation from the genomewide profile. Histogram of Euclidean distances between GC-normalized RSCU values of single genes and the genomewide profile of P. pacificus. For all orphans and non orphans, the frequency of genes in each Euclidean distance bin is shown. [file 1471-2148-11-239-S1.PDF]
